# Regulation of Innate Immune Response to Fungal Infection in *Caenorhabditis elegans* by SHN-1/SHANK

**DOI:** 10.4014/jmb.2006.06025

**Published:** 2020-09-11

**Authors:** Lingmei Sun, Huirong Li, Li Zhao, Kai Liao

**Affiliations:** 1 Department of Pharmacology, Medical School of Southeast University, Nanjing 20009, P.R. China; 2 Key Laboratory of Developmental Genes and Human Disease in Ministry of Education, Medical School, Southeast University, Nanjing 10009, P.R. China; 3 Department of Pathology and Pathophysiology, Medical School of Southeast University, Nanjing 210009, P.R. China

**Keywords:** SHN-1, innate immunity, neurotransmitter, *Candida albicans*, *Caenorhabditis elegans*

## Abstract

In *Caenorhabditis elegans*, SHN-1 is the homologue of SHANK, a scaffolding protein. In this study, we determined the molecular basis for SHN-1/SHANK in the regulation of innate immune response to fungal infection. Mutation of *shn-1* increased the susceptibility to *Candida albicans* infection and suppressed the innate immune response. After *C. albicans* infection for 6, 12, or 24 h, both transcriptional expression of *shn-1* and SHN-1::GFP expression were increased, implying that the activated SHN-1 may mediate a protection mechanism for *C. elegans* against the adverse effects from fungal infection. SHN-1 acted in both the neurons and the intestine to regulate the innate immune response to fungal infection. In the neurons, GLR-1, an AMPA ionotropic glutamate receptor, was identified as the downstream target in the regulation of innate immune response to fungal infection. GLR-1 further positively affected the function of SER-7-mediated serotonin signaling and antagonized the function of DAT-1-mediated dopamine signaling in the regulation of innate immune response to fungal infection. Our study suggests the novel function of SHN-1/SHANK in the regulation of innate immune response to fungal infection. Moreover, our results also denote the crucial role of neurotransmitter signals in mediating the function of SHN-1/SHANK in regulating innate immune response to fungal infection.

## Introduction

Innate immune response is an important defense mechanism in animals and human beings against microbial infection. A large amount of evidence has implied the evolutionarily conserved property of the innate immune system between vertebrates, such as mammals, and invertebrates, such as *Caenorhabditis elegans* [1, 2]. Upon infection, *C. elegans* can potentially activate an inducible innate immune system or avoid pathogens [3-5]. After infection with certain bacterial or fungal pathogens, *C. elegans* can exhibit a rapid innate immune response as has been observed in other organisms [6-9]. An important advantage of the *C. elegans*-pathogen pathogenesis system is that it can reflect the different stages of mammalian infection despite the few commonalities between the known components of *C. elegans* and mammalian immunity; general defense strategies, such as recognition, signaling, and response, are conserved [10, 11]. Thus, the use of *C. elegans* as a powerful in vivo model animal for the study of infection and innate immune response to *Candida albicans* has the potential to teach us about the evolutionary origins of immunity and may reveal as yet uncharacterized aspects of mammalian defenses against infection [9].

*C. albicans* is the most common fungal pathogen of human beings in clinical settings [12-14]. Under normal circumstances, *C. albicans* is harmless to human beings. However, if the immune system is weakened or competing bacterial flora are eliminated in hosts, it can invade host tissues, colonize the host gastrointestinal tract, and even lead to life-threatening infections [7, 15, 16]. After exposure to *C. albicans*, some putative antimicrobial genes, including *abf-2*, *cnc-4*, *cnc-7*, and *fipr-22/23*, are induced and activated [17, 18]. Several important signaling pathways, such as the insulin and p38 mitogen-activated protein kinase signaling pathways, have been identified as being required for the regulation of innate immune response to *C. albicans* infection [17, 19, 20]. Nevertheless, the molecular mechanisms underlying innate immune response to *C. albicans* infection in *C. elegans* remain largely unclear.

In mammals, for example, rats, SHANK proteins have important functions in mediating proper protein localization at the postsynaptic density (PSD), and they form a complex by interacting with a variety of membrane and cytoplasmic proteins, such as GPAK, a PSD protein [21, 22]. The SHANK-associated RH domain-interacting protein SHARPIN is a key regulator of immune and inflammatory responses [21]. Moreover, SHANK regulates the proper localization of receptor proteins, including glutamate receptors, that are necessary to induce T-cell activation and modulate immune function [23, 24]. In *C. elegans*, SHN-1 is the homologue of SHANK, a scaffolding protein containing the PDZ (PSD-95, Dlg, and ZO-1) domain [25]. Herein, we determined the molecular basis of the involvement of SHN-1/SHANK in the regulation of innate immune response to fungal infection by using the *C. elegans*-*C. albicans* pathogenesis system to understand the role of SHN-1/SHANK in the regulation of innate immunity. Our data demonstrated the tissue-specific activities of SHN-1/SHANK in neurons and in intestine in the regulation of innate immune response to fungal infection. We further identified the specific signaling cascades mediated by SHN-1/SHANK in neurons in the regulation of innate immune response to fungal infection. Our study will be helpful for understanding the molecular mechanisms underlying the role of SHN-1/SHANK in the regulation of innate immunity after pathogen infection.

## Materials and Methods

### Strains and Media

*C. elegans* was maintained on nematode growth medium (NGM, 3 g/l NaCl, 2.5 g/l polypeptone, 5 mg/l cholesterol, 1 mM CaCl_2_, 1 mM MgSO_4_, 25 mM KH_2_PO_4_, and 17 g/L agar) plates seeded with *Escherichia coli* OP50 at 20°C as described [26]. *C. elegans* strains used in this study were wild-type N2, mutants of *shn-1(ok1241)*, *egl-19(tm8983)*, *mgl-2(tm355)*, *dyn-1(ky51)*, *glr-1(n2461)*, *zag-1(ok214)*, *dat-1(ok157)*, *ser-7(tm1325)*, *dop-1(vs101)*, and *glr-1(n2461)dat-1(ok157)*, and transgenic strains of *Ex*(P*shn-1*-*shn-1::GFP*), *shn-1(ok1241)Ex*(P*ges*-1-*shn-1*), *shn-1(ok1241)Ex*(P*myo*-2-*shn-1*), *shn-1(ok1241)Ex*(P*unc*-14-*shn-1*), *Is*(P*ges*-1-*shn-1*), *Is*(P*unc*-14-*shn-1*), *Is*(P*unc*-14-*shn-1*);*egl-19(tm8983)*, *Is*(P*unc*-14-*shn-1*);*glr-1(n2461)*, *Is*(P*unc*-14-*glr-1*), and *Is*(P*unc*-14-*glr-1*);*ser-7(tm1325)*. Age-synchronous populations of *C. elegans* (4 days old post egg lay) were used in this study. *C. albicans* SC5314 (wild-type strain) was grown in liquid yeast extract-peptone-dextrose (YPD, 10 g/l yeast extract, 20 g/l peptone, 20 g/l dextrose) broth or on brain heart infusion (BHI, 37 g/l BHI) agar containing kanamycin (45 μg/ml) at 30°C. Bacteria were grown in Luria Broth (LB, 10 g/l tryptone, 5 g/l yeast extract, 10 g/l NaCl).

### *C. elegans* Survival Assay

*C. elegans* survival analysis was performed as previously described [27]. *C. albicans* wild-type strain SC5314 was seeded on the plates containing brain heart infusion (BHI) and kanamycin (45 μg/ml). Age-synchronous populations of *C. elegans* were washed from NGM plates with M9 buffer (3 g/l KH_2_PO_4_, 6 g/l Na_2_HPO_4_, 5 g/l NaCl, 1mM MgSO_4_), and then added to the center of the *C. albicans* lawns for 4 h at 25°C. The fungal infection was started by adding 60 animals to each plate. After that, the examined *C. elegans* were transferred into a single well of a tissue culture plate (Corning, Inc.) containing 2 ml of liquid medium (80% M9 buffer and 20% BHI) and kanamycin (45 μg/ml). In the assay plates, fluoro-29-deoxyuridine (FUdR, 75 mg/ml) was added to prevent the growth of progeny. *C. elegans* were scored for dead or live every 24 h. *C. elegans* would be scored as dead if no response was detected after prodding with a platinum wire. Three replicates were analyzed for each experiment.

### CFU Assay of *C. albicans*

The number of *C. albicans* CFU in *C. elegans* was quantified as described previously [20, 28]. *C. elegans* were infected with *C. albicans* lawns for 24 h. After washing with sterile M9 buffer for five times to remove the surface *C. albicans*, each group of 50 *C. elegans* was disrupted using a homogenizer, and then plated on a YPD agar containing kanamycin (45 μg/ml), ampicillin (100 μg/ml), and streptomycin (100 μg/ml). The plates were incubated for 48 h at 37°C. *C. albicans* colonies were counted to determine the CFU per nematode. Ten replicates were analyzed for each experiment.

### Quantitative Reverse Transcription-Polymerase Chain Reaction (qRT-PCR)

Total RNA was extracted from *C. elegans* according to the manufacturer’s protocol in an RNeasy Mini Kit (Qiagen). Purity and concentration of RNAs were analyzed by the ratio of OD 260/280 in a spectrophotometer. cDNA was synthesized in a 12.5 μl reaction volume containing 625 ng total RNA, 0.5 mM reverse-transcript primers, 50 mM Tris-HCl, 75 mM KCl, 3 mM MgCl_2_, 10 mM dithiothreitol, 20 units of ribonuclease inhibitor, and 100 units of reverse transcriptase (Takara, China). The reaction mixture was incubated at 25°C for 5 min, followed by 42°C for 60 min. The reverse transcriptase was inactivated at 70°C for 15 min. Transcriptional quantification was determined by real-time PCR in an ABI 7500 real-time PCR system using Evagreen (Biotium, USA). The putative antimicrobial genes were selected as described [18]. The final results were expressed as relative expression ratio between targeted genes including the antimicrobial genes and reference *tba-1* gene encoding a tubulin protein. All reactions were performed in triplicate. The related primers for targeted genes and reference gene were shown in Table S1.

### DNA Constructs and Germline Transformation

To generate entry vector carrying promoter sequence, promoter region for *shn-1* gene, *ges-1* gene specially expressed in the intestine, *myo-2* gene specially expressed in the pharynx, or *unc-14* gene specially expressed in the neurons was amplified by PCR from wild-type *C. elegans* genomic DNA. The promoter fragment was inserted into the GFP expression vector pPD95_77 in the sense orientation. *shn-1/C33B4.3c*, or *glr-1/C06E1.4* cDNA was amplified by PCR, and then inserted into the corresponding entry vector carrying the *shn-1*, *ges-1*, *myo-2*, or *unc-14* promoter sequence. The designed primers for DNA construct generation were shown in Table S2. Germline transformation was performed by coinjecting a testing DNA (10-40 μg/ml) and a marker DNA of P*dop-1::rfp* or *unc-119*(+) (60 μg/ml) into the gonad of *C. elegans* as described previously [29].

### Fluorescence Microscope

The transgenic strain *Ex*(P*shn-1*-*shn-1::GFP*) was infected with *C. albicans* lawns for 0, 12, or 48 h. After washing with sterile M9 buffer for three times, 50 worms were picked randomly to detect the GFP fluorescence. We used ImageJ software to analyze the images and calculated the relative fluorescence intensity.

### RNAi Assay

We fed the *C. elegans* with *E. coli* strain HT115 (DE3) expressing double-stranded RNA that is homologous to a certain gene as described [4]. *E. coli* HT115 (DE3) grown in LB broth containing ampicillin (100 μg/ml) at 37°C overnight was plated onto NGM plates containing ampicillin (100 μg/ml) and isopropyl 1-thio-β-D-galactopyranoside (IPTG, 5 mM). L1 larvae were placed on the RNAi plates for 2 days until *C. elegans* became gravid. The gravid adults were transferred to fresh RNAi-expressing bacterial lawns to let them lay eggs so as to obtain the second generation of RNAi population. The eggs were allowed to develop into *C. elegans* for the subsequent assays. The RNAi efficiency was confirmed by qRT-PCR (data not shown).

### Statistical Analysis

Data in the present study were presented as means ± standard deviation (SD). Graphs were prepared using Microsoft Excel software (Microsoft Corp., USA). Statistical analysis was performed using SPSS 12.0 software (SPSS Inc., USA). Differences between groups were determined using analysis of variance (ANOVA), and probability levels of 0.05 and 0.01 were considered statistically significant. In the *C. elegans* survival assay, the Kaplan-Meier method was used to calculate survival fractions and a statistically significant p value was calculated using a log-rank test.

## Results

### Effect of *C. albicans* Infection on *shn-1* Expression

**Fig. 1:**
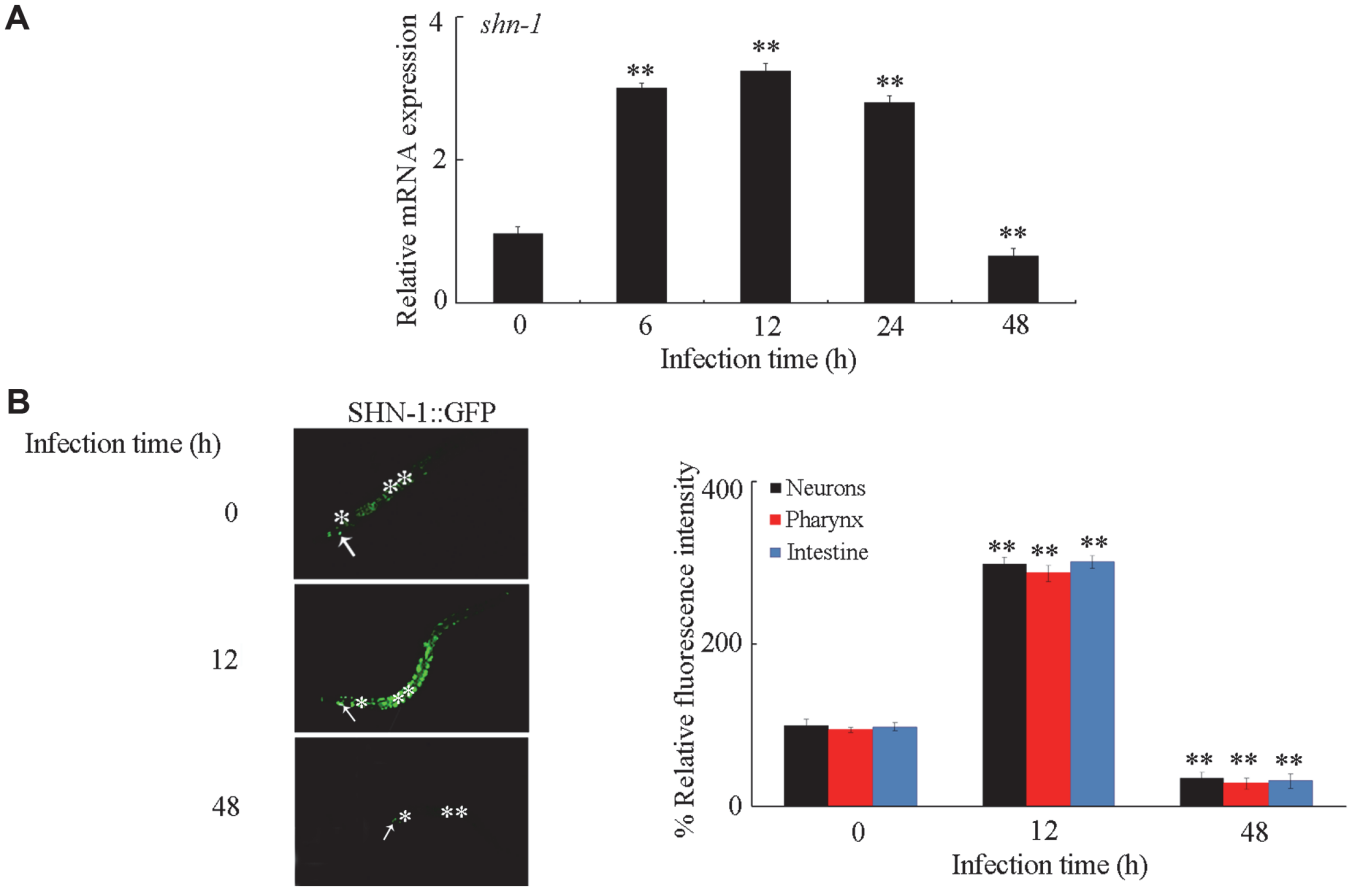
Response of *shn-1* to *C. albicans* infection. (**A**) Effect of *C. albicans* infection on transcriptional expression of *shn-1*. The reactions were performed in triplicate. (**B**) Effect of *C. albicans* infection on expression of SHN-1::GFP. Arrowheads indicate the neurons. Pharynx (*) and intestine (**) were also indicated. Thirty animals were examined. Bars represent mean SD. **p < 0.01 vs 0 h.

We first investigated the effect of *C. albicans* SC5314 infection on the transcriptional expression of *shn-1*. After *C. albicans* infection for 6 h, we observed a significant increase in the transcriptional expression of *shn-1* (Fig. 1A). The increase in the transcriptional expression of *shn-1* could be further detected in wild-type *C. elegans* after *C. albicans* infection for 12 or 24 h (Fig. 1A). By contrast, the transcriptional expression of *shn-1* in wild-type *C. elegans* after *C. albicans* infection for 48 h was significantly decreased relative to that at pre-infection (Fig. 1A).

In *C. elegans*, SHN-1 is expressed in various tissues, including neurons, the pharynx, and the intestine [25]. By using the transgenic strain *Ex*(P*shn-1*-*shn-1::GFP*), we further revealed that SHN-1::GFP in neurons, the pharynx, and the intestine was significantly increased after *C. albicans* infection for 12 h (Fig. 1B). By contrast, after *C. albicans* infection for 48 h, the expression of SHN-1::GFP in neurons, the pharynx, or the intestine was significantly decreased (Fig. 1B).

### Loss-of-Function Mutation of *shn-1* Suppressed Innate Immune Response to *C. albicans* Infection

**Fig. 2:**
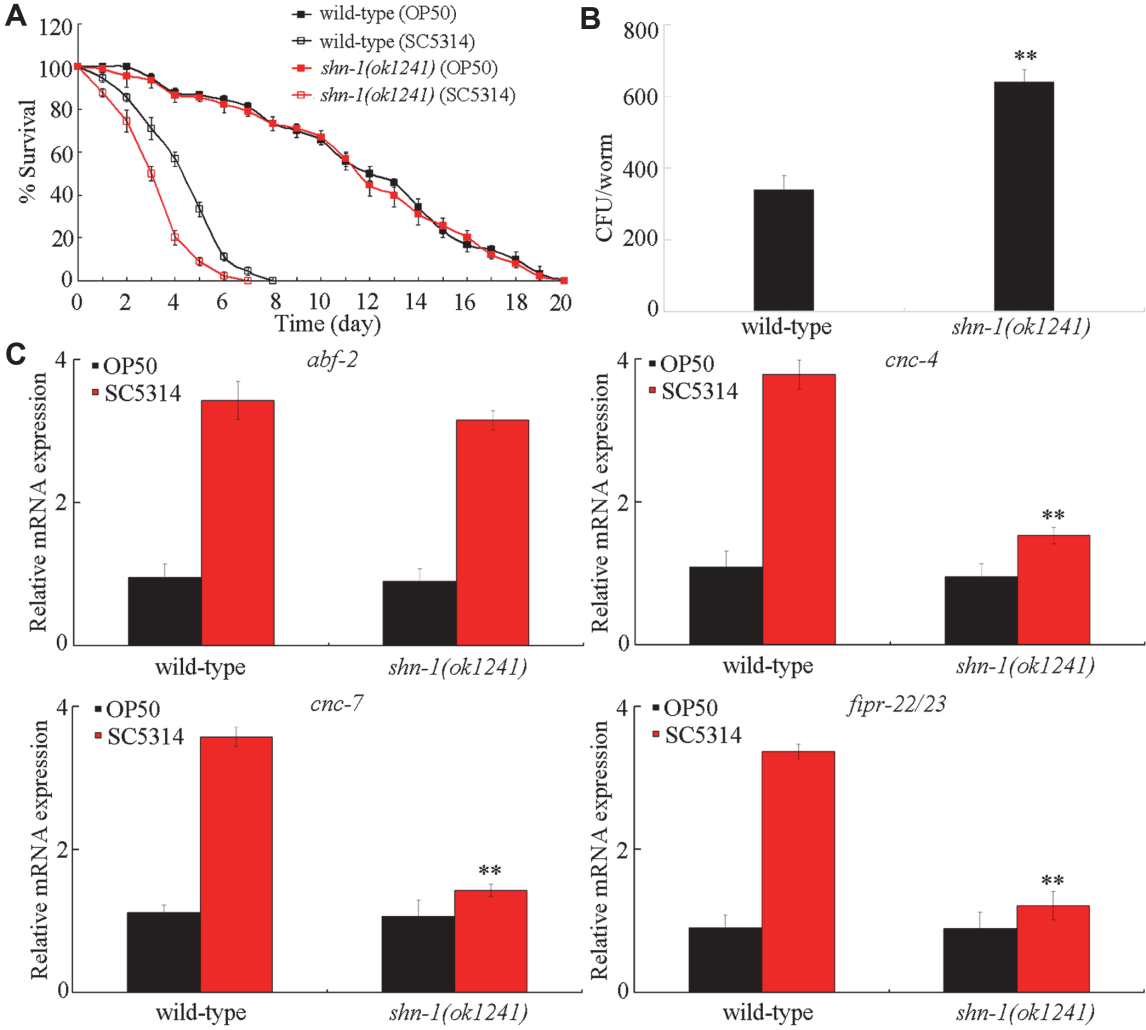
Function of SHN-1 in the regulation of innate immune response to *C. albicans* infection. (**A**) Effect of *shn-1* mutation on survival in *C. albicans*-infected *C. elegans*. Statistical comparison of the survival plots indicates that, after *C. albicans* infection, the survival of *shn-1(ok1241)* mutant was significantly different from that of wild-type *C. elegans* (*p* < 0.0001). Under normal conditions, the survival of *shn-1(ok1241)* mutant was not significantly different from that of wild-type *C. elegans* (*p* = 0.9875). Three replicates were performed. (**B**) Effect of *shn-1* mutation on CFU of *C. albicans* in *C. elegans*. *C. elegans* were infected with *C. albicans* lawns for 24 h. (**C**) Effects of *shn-1* mutation on expression levels of putative antimicrobial genes in *C. albicans*-infected *C. elegans*. *C. elegans* were infected with *C. albicans* lawns for 24 h. The reactions were performed in triplicate. Bars represent mean ± SD. **p < 0.01 vs wild-type.

Under normal conditions, the loss-of-function mutation of *shn-1* does not affect longevity (Fig. 2A). However, after *C. albicans* infection, the loss-of-function mutation of *shn-1* caused the significant decrease in the survival of *C. elegans* (Fig. 2A). Meanwhile, the loss-of-function mutation of *shn-1* increased the number of colony-forming units (CFU) of *C. albicans* in *C. elegans* (Fig. 2B). Therefore, the loss-of-function mutation of *shn-1* might increase susceptibility to *C. albicans* infection.

We further utilized *abf-2*, *cnc-4*, *cnc-7*, and *fipr-22/23* as putative antimicrobial genes to investigate the potential effect of the loss-of-function mutation of *shn-1* on innate immune response to *C. albicans* infection. After *C. albicans* infection, the putative antimicrobial genes *abf-2*, *cnc-4*, *cnc-7*, and *fipr-22/23* were induced in wild-type *C. elegans* [18]. Under normal conditions, the loss-of-function mutation of *shn-1* did not significantly influence the expression levels of the examined putative antimicrobial genes (Fig. 2C). After *C. albicans* infection for 24 h, although the loss-of-function mutation of *shn-1* did not affect the transcriptional expression of *abf-2*, we observed a significant reduction in the expression levels of *cnc-4*, *cnc-7*, and *fipr-22/23* in the *C. albicans*-infected *shn-1* mutant compared with those in the *C. albicans*-infected wild-type *C. elegans* (Fig. 2C). These results suggested that SHN-1 might be required for the regulation of innate immune response to fungal infection.

### Tissue-Specific Activities of SHN-1 in the Regulation of Innate Immune Response to *C. albicans* Infection

**Fig. 3:**
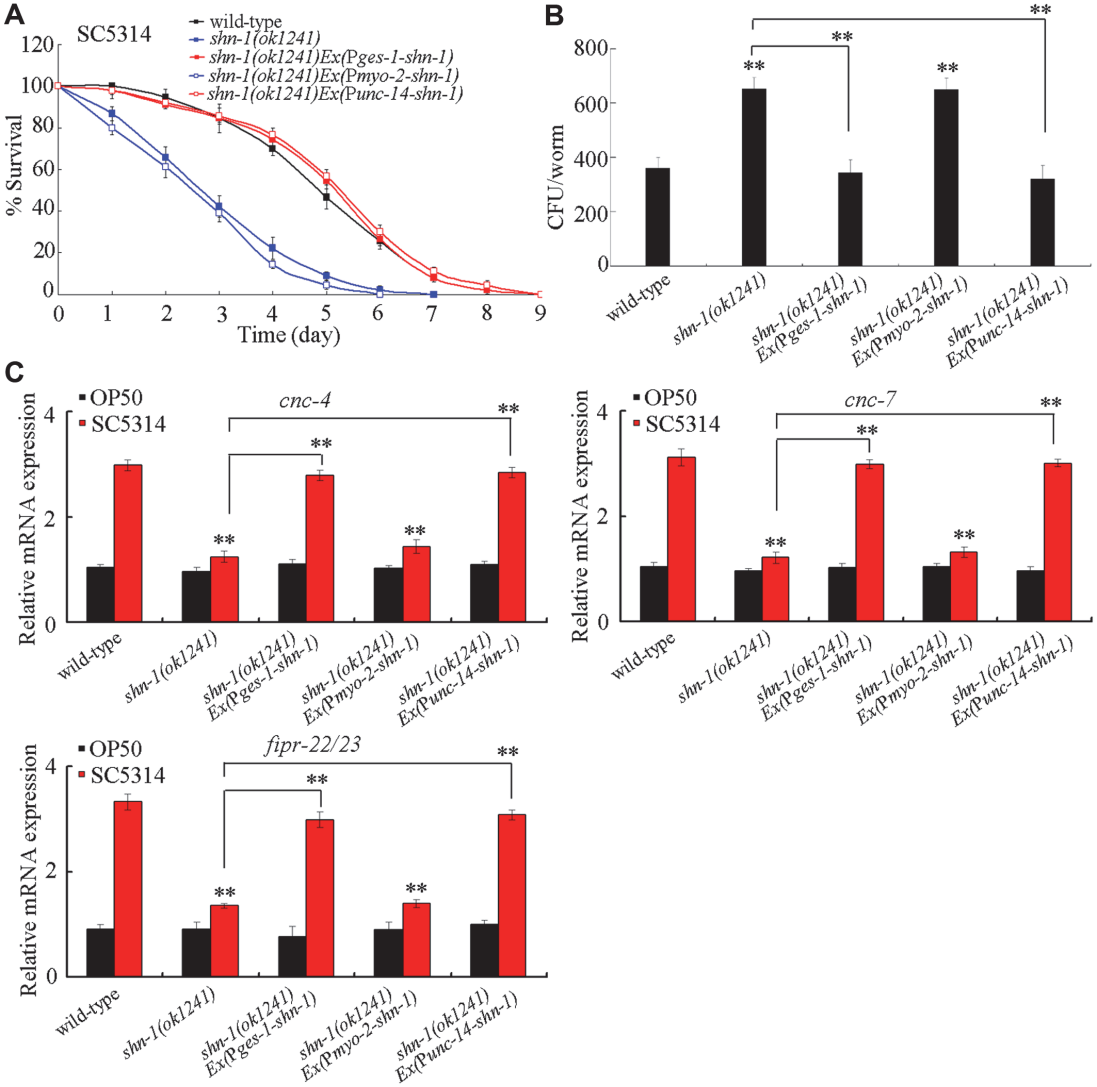
Tissue-specific activities of SHN-1 in the regulation of innate immunity. (**A**) Tissue-specific activities of SHN-1 in the regulation of survival in *C. albicans*-infected *C. elegans*. Statistical comparisons of the survival plots indicate that, after *C. albicans* infection, the survival of *shn-1(ok1241)Ex*(P*ges*-1-*shn-1*) (*p* < 0.0001) or *shn-1(ok1241)Ex*(P*unc*-14-*shn-1*) (*p* < 0.0001) was significantly different from that of *shn-1(ok1241)*, and the survival of *shn-1(ok1241)Ex*(P*myo*-2-*shn-1*) (*p* = 0.9719) was not significantly different from that of *shn-1(ok1241)*. Three replicates were performed. (**B**) Tissue-specific activities of SHN-1 in the regulation of CFU of *C. albicans* in *C. elegans*. *C. elegans* were infected with *C. albicans* lawns for 24 h. (**C**) Tissuespecific activities of SHN-1 in the regulation of expression levels of putative antimicrobial genes in *C. albicans*-infected *C. elegans*. *C. elegans* were infected with *C. albicans* lawns for 24 h. The reactions were performed in triplicate. Bars represent mean ± SD. **p < 0.01 vs wild-type (if not specially indicated).

We further examined the tissue-specific activities of SHN-1 in the regulation of innate immune response to fungal infection. After *C. albicans* infection, the intestinal or neuronal expression of *shn-1* significantly increased the survival of the *shn-1(ok1241)* mutant and decreased the CFU of *C. albicans* in the *shn-1(ok1241)* mutant (Figs. 3A and 3B). By contrast, after *C. albicans* infection, the expression of *shn-1* in the pharynx did not significantly affect the survival and CFU of *C. albicans* in the *shn-1(ok1241)* mutant (Figs. 3A and 3B).

Considering the fact that the *shn-1* mutant could decrease the expression levels of *cnc-4*, *cnc-7*, and *fipr-22/23* in *C. albicans*-infected *C. elegans* as indicated above, we further selected these putative antimicrobial genes to examine the tissue-specific activities of SHN-1 in the regulation of innate immune response to *C. albicans* infection. After *C. albicans* infection, the intestinal or neuronal expression of *shn-1* significantly increased the expression levels of *cnc-4*, *cnc-7*, and *fipr-22/23* in the *shn-1(ok1241)* mutant (Fig. 3C). By contrast, after *C. albicans* infection, the expression of *shn-1* in the pharynx did not significantly influence the expression levels of *cnc-4*, *cnc-7*, and *fipr-22/23* in the *shn-1(ok1241)* mutant (Fig. 3C). These results suggested that SHN-1 could act in the intestine and neurons to regulate innate immune response to *C. albicans* infection.

### Neuronal or Intestinal Overexpression of SHN-1-Enhanced Antifungal Immunity against *C. albicans* Infection

**Fig. 4:**
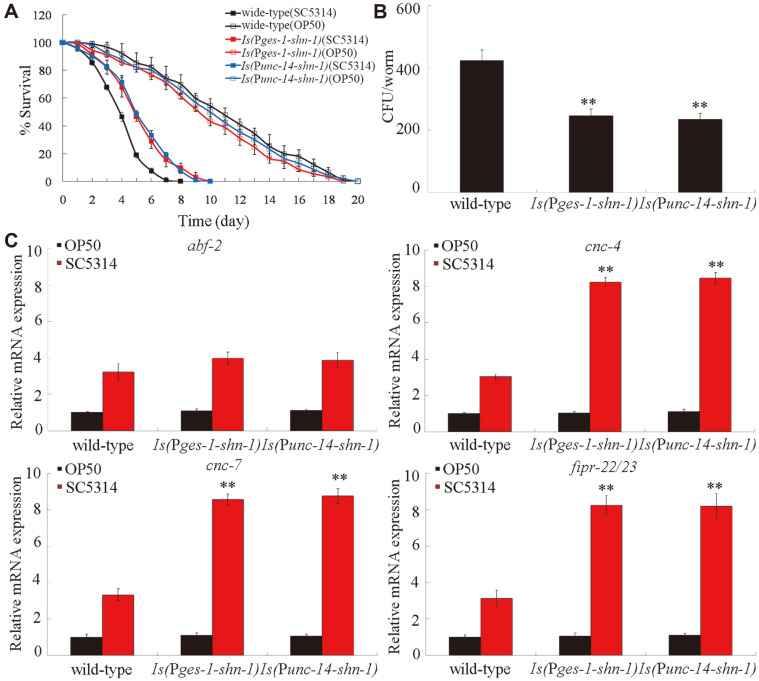
Effect of neuronal or intestinal overexpression of SHN-1 on innate immune response to *C. albicans* infection. (**A**) Effect of neuronal or intestinal overexpression of SHN-1 on survival in *C. albicans*-infected *C. elegans*. Statistical comparisons of the survival plots indicate that, after *C. albicans* infection, the survival of *C. elegans* with *shn-1* overexpression in the neurons or the intestine was significantly different from that of wild-type (*p* < 0.0001). Three replicates were performed. (**B**) Effect of neuronal or intestinal overexpression of SHN-1 on CFU of *C. albicans* in *C. elegans*. *C. elegans* were infected with *C. albicans* lawns for 24 h. (**C**) Effect of neuronal or intestinal overexpression of SHN-1 on expression levels of putative antimicrobial genes in *C. albicans*-infected *C. elegans*. *C. elegans* were infected with *C. albicans* lawns for 24 h. The reactions were performed in triplicate. Bars represent mean ± SD. **p < 0.01 vs wild-type.

On the basis of the identified tissue-specific activities of SHN-1 in the regulation of innate immune response to fungal infection, we next examined the effect of the neuronal or intestinal overexpression of SHN-1 on innate immune response to *C. albicans* infection. Under normal conditions, the overexpression of intestinal or neuronal SHN-1 did not affect the longevity of *C. elegans* (Fig. 4A). After *C. albicans* infection, the neuronal or intestinal overexpression of SHN-1 increased survival and decreased the CFU of *C. albicans* in *C. elegans* (Figs. 4B and 4C). With the aid of putative antimicrobial genes, we further found that the neuronal or intestinal overexpression of SHN-1 significantly increased the expression levels of *cnc-4*, *cnc-7*, and *fipr-22/23* after *C. albicans* infection, although the neuronal or intestinal overexpression of SHN-1 did not obviously influence the expression level of *abf-2* after *C. albicans* infection (Fig. 4C). Under normal conditions, the neuronal or intestinal overexpression of SHN-1 did not significantly influence the expression levels of the examined putative antimicrobial genes (Fig. 4C). Therefore, the neuronal or intestinal overexpression of SHN-1 might enhance antifungal immunity to fungal infection.

### Identification of the Potential Downstream Targets of SHN-1 in the Regulation of Innate Immune Response to *C. albicans* Infection

**Fig. 5:**
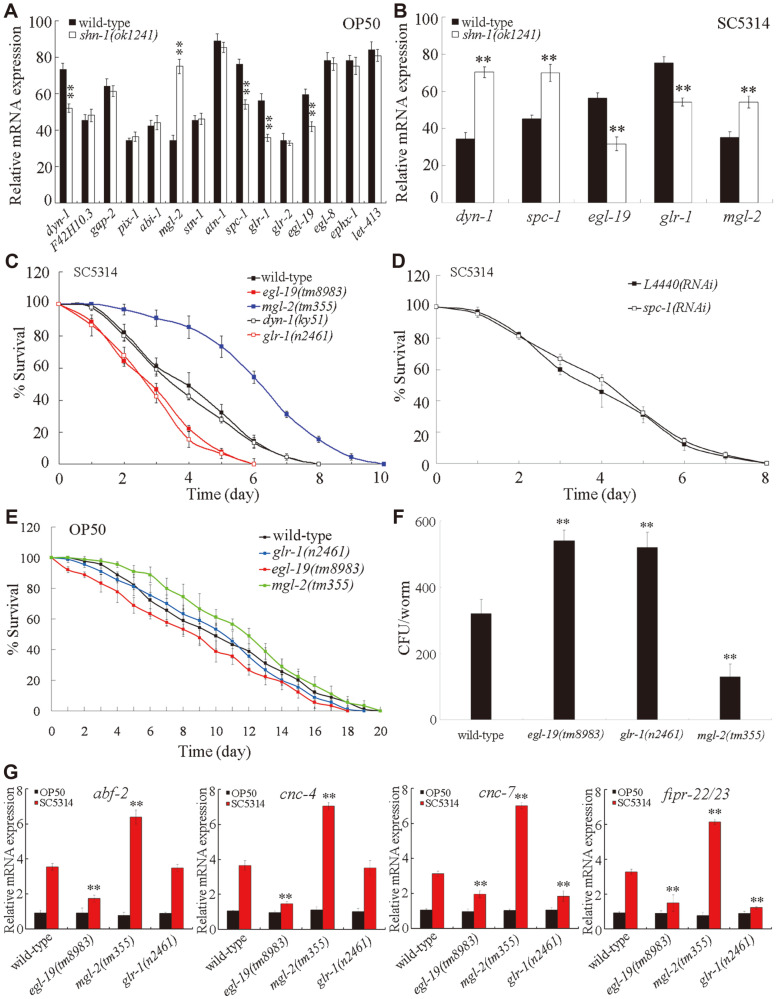
Identification of potential downstream targets of SHN-1 in the regulation of innate immune response to *C. albicans* infection. (**A**) Effect of *shn-1* mutation on expression levels of candidate targeted genes under normal conditions. (**B**) Effect of *shn-1* mutation on expression levels of *dyn-1*, *spc-1*, *egl-19*, *glr-1*, and *mgl-2* after *C. albicans* infection. (**C**) Effect of *egl-19*, *mgl-2*, *dyn-1*, or *glr-1* mutation on survival of *C. albicans* infected *C. elegans*. Statistical comparisons of the survival plots indicate that, after *C. albicans* infection, the survival of *egl-19(tm8983)*, *mgl-2(tm355)*, or *glr-1(n2461)* was significantly different from that of wild-type (*p* < 0.0001), and the survival of *dyn-1(ky51)* was not significantly different from that of wild-type (*p* = 0.9182). Three replicates were performed. (**D**) Effect of RNAi knockdown of *spc-1* on survival of *C. albicans* infected *C. elegans*. L4440(RNAi), empty vector RNAi. Statistical comparison of the survival plots indicates that, after *C. albicans* infection, the survival of *spc-1*(RNAi) was not significantly different from that of L4440(RNAi). (**E**) Mutation of *glr-1*, *egl-19*, or *mgl-2* did not affect the longevity under normal conditions. Statistical comparisons of the survival plots indicate that, under normal conditions, the survival of *egl-19(tm8983)* (*p* = 0.9486), *mgl-2(tm355)* (*p* = 0.9122), or *glr-1(n2461)* (*p* = 0.9712) was not significantly different from that of wild-type. (**F**) Effect of *glr-1*, *egl-19*, or *mgl-2* mutation on CFU of *C. albicans* in *C. elegans*. *C. elegans* were infected with *C. albicans* lawns for 24 h. (**G**) Effect of *glr-1*, *egl-19*, or *mgl-2* mutation on expression levels of putative antimicrobial genes in *C. albicans* infected *C. elegans*. *C. elegans* were infected with *C. albicans* lawns for 24 h. The reactions were performed in triplicate. Bars represent mean ± SD. **p < 0.01 vs wild-type.

In mammals, more than 30 synaptic proteins can interact with SHANK proteins [30]. We searched the homologues of these interacting proteins in *C. elegans* (GAP-2/SynGAP, PIX-1/PIX, ABI-1/Abi1, STN-1/syntrophin, ATN-1/ACTN, GLR-2/AMPA ionotropic glutamate receptor, EGL-8/PLC-β, EPHX-1/ARHGEF, F42H10.3/NEBL, LET-413/Densin, DYN-1/Dynamin, SPC-1/α spectrin, GLR-1/AMPA ionotropic glutamate receptor, EGL-19/voltage-gated calcium channel, and MGL-2/metabotropic glutamate receptor) as the possible candidate targets of SHN-1. Under normal conditions, the loss-of-function mutation of *shn-1* did not significantly affect the expression levels of F42H10.3, gap-2, pix-1, abi-1, stn-1, atn-1, *glr-2*, egl-8, ephx-1, and let-413, whereas the loss-of-function mutation of *shn-1* significantly decreased the expression of *dyn-1*, *spc-1*, *glr-1*, and *egl-19* and increased the expression of *mgl-2* (Fig. 5A). Moreover, after *C. albicans* infection, the loss-of-function mutation of *shn-1* significantly decreased the expression levels of *dyn-1*, *spc-1*, *glr-1*, and *egl-19* and increased the expression of *mgl-2* (Fig. 5B). These results implied that DYN-1, SPC-1, GLR-1, EGL-19, and MGL-2 might be the downstream targets of SHN-1 in the regulation of innate immune response to *C. albicans* infection.

We further determined the potential roles of DYN-1, SPC-1, GLR-1, EGL-19, and MGL-2 in the regulation of *C. albicans* infection. After *C. albicans* infection, the mutation of *egl-19* or *glr-1* decreased survival, and the mutation of *mgl-2* increased the survival of *C. elegans* (Fig. 5C). By contrast, after *C. albicans* infection, the mutation of *dyn-1* did not significantly affect survival (Fig. 5C). Meanwhile, after *C. albicans* infection, the RNA interference (RNAi) knockdown of *spc-1* did not significantly influence survival (Fig. 5D). Under normal conditions, the lifespan of the *glr-1(n2461)*, *egl-19(tm8983)*, or *mgl-2(tm355)* mutant was similar to that of wild-type (Fig. 5E). Moreover, the mutation of *egl-19* or *glr-1* increased the CFU of *C. albicans*, whereas the mutation of *mgl-2* decreased the CFU of *C. albicans* (Fig. 5F). With the aid of putative antimicrobial genes, we further found that the mutation of *egl-19* could significantly decrease the expression levels of *abf-2*, *cnc-4*, *cnc-7*, and *fipr-22/23* after *C. albicans* infection, and the mutation of *glr-1* could significantly decrease the expression levels of *cnc-7* and *fipr-22/23* after *C. albicans* infection (Fig. 5G). By contrast, the mutation of *mgl-2* could significantly increase the expression levels of *abf-2*, *cnc-4*, *cnc-7*, and *fipr-22/23* after *C. albicans* infection (Fig. 5G). Therefore, GLR-1, EGL-19, and MGL-2 might act as the potential downstream targets of SHN-1 in the regulation of innate immune response to fungal infection.

### Identification of the Downstream Targets of Neuronal SHN-1 in the Regulation of Innate Immune Response to *C. albicans* Infection

**Fig. 6:**
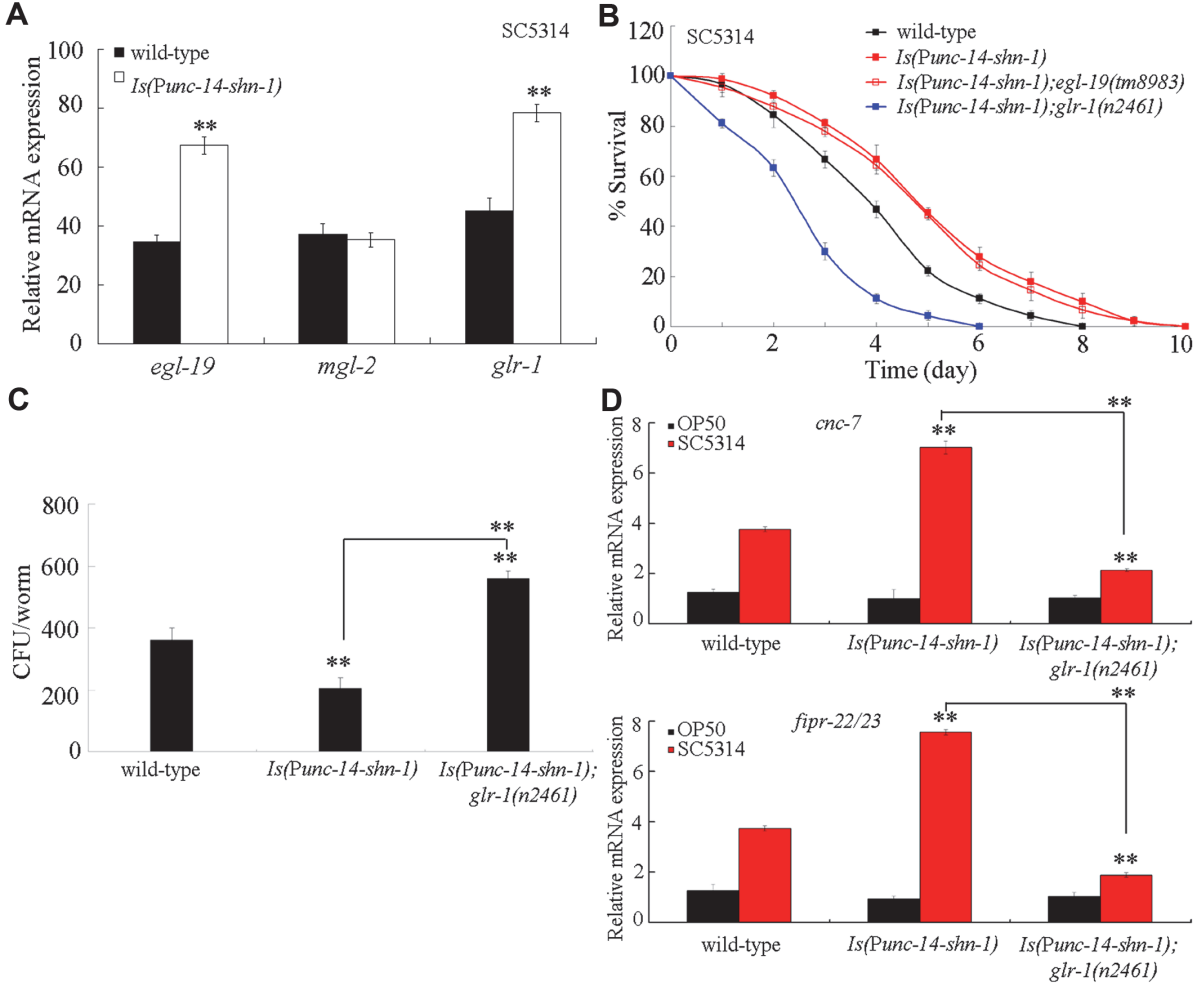
Identification of downstream targets of neuronal SHN-1 in the regulation of innate immune response to *C. albicans* infection. (**A**) Effect of overexpression of neuronal SHN-1 on expression levels of *egl-19*, *mgl-2*, and *glr-1* genes after *C. albicans* infection. (**B**) Effect of *egl-19* or *glr-1* mutation on survival of *C. elegans* overexpressing neuronal SHN-1 after *C. albicans*-infection. Statistical comparisons of the survival plots indicate that, after *C. albicans*-infection, the survival of *Is*(P*unc*-14-*shn-1*);*glr-1(n2461)* was significantly different from that of *Is*(P*unc*-14-*shn-1*) (*p* < 0.0001), and the survival of *Is*(P*unc*-14-*shn-1*);*egl-19(tm8983)* was not significantly different from that of *Is*(P*unc*-14-*shn-1*) (*p* = 0.9182). Three replicates were performed. (**C**) Effect of *glr-1* mutation on CFU of *C. albicans* in *C. elegans* overexpressing neuronal SHN-1. *C. elegans* were infected with *C. albicans* lawns for 24 h. (**G**) Effect of *glr-1* mutation on expression levels of putative antimicrobial genes in *C. elegans* overexpressing neuronal SHN-1 after *C. albicans*-infection. *C. elegans* were infected with *C. albicans* lawns for 24 h. The reactions were performed in triplicate. Bars represent mean ± SD. **p < 0.01 vs wild-type (if not specially indicated).

We further attempted to identify the downstream targets of neuronal SHN-1 in the regulation of innate immune response to fungal infection. In *C. elegans*, *egl-19*, *glr-1*, and *mgl-2* can be expressed in neurons [31-33]. Among the dysregulation of genes induced by *shn-1* mutation after *C. albicans* infection, we found that the neuronal overexpression of SHN-1 significantly increased the expression levels of *egl-19* and *glr-1* after *C. albicans* infection (Fig. 6A). By contrast, after *C. albicans* infection, the neuronal overexpression of SHN-1 did not significantly affect the expression level of *mgl-2* (Fig. 6A).

We next focused on EGL-19 and GLR-1 to examine their interaction with neuronal SHN-1 in the regulation of innate immune response to fungal infection. In the transgenic strain overexpressing neuronal SHN-1, we found that the *glr-1* mutation, but not the *egl-19* mutation, could significantly decrease the survival of *C. albicans*-infected *C. elegans* (Fig. 6B). Moreover, the mutation of *glr-1* significantly increased the CFU of *C. albicans* in *C. elegans* overexpressing neuronal SHN-1 (Fig. 6C). Considering that the overexpression of neuronal SHN-1 could affect the expressions levels of *cnc-4*, *cnc-7*, and *fipr-22/23* and *glr-1* mutation could affect the expressions levels of *cnc-7* and *fipr-22/23*, we selected *cnc-7* and *fipr-22/23* as putative antimicrobial genes to investigate the interaction between GLR-1 and SHN-1 in the regulation of innate immune response to fungal infection. We found that the mutation of *glr-1* significantly decreased the expression levels of *cnc-7* and *fipr-22/23* in *C. elegans* overexpressing neuronal SHN-1 after *C. albicans* infection (Fig. 6D). Therefore, GLR-1 might act as the downstream target of neuronal SHN-1 in the regulation of *C. albicans* infection.

### Identification of the Downstream Targets of GLR-1 in the Regulation of Innate Immune Response to *C. albicans* Infection

**Fig. 7:**
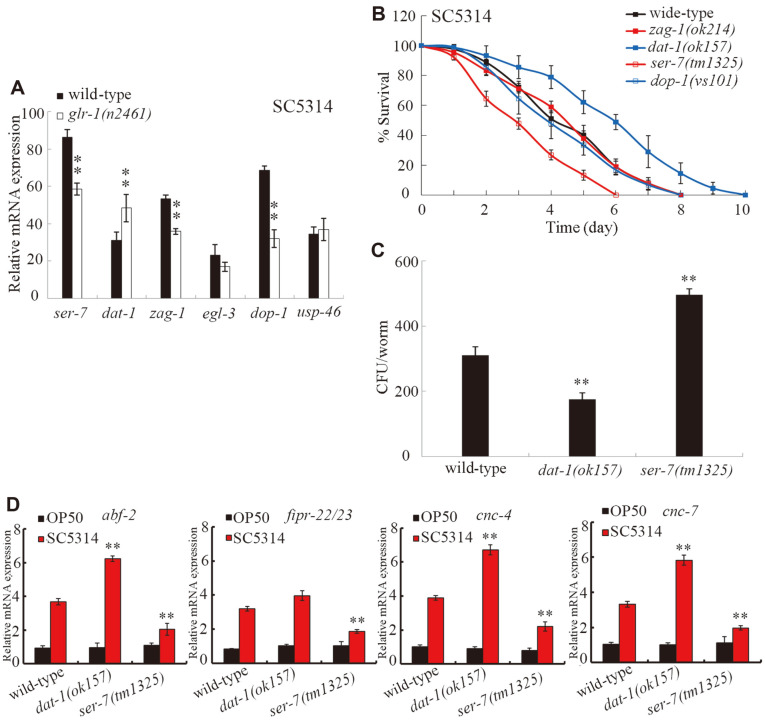
Identification of downstream targets of GLR-1 in the regulation of innate immune response to *C. albicans* infection. (**A**) Effect of *glr-1* mutation on the expression levels of *ser-7*, *dat-1*, *zag-1*, *egl-3*, *dop-1*, and *usp-46* genes after *C. albicans* infection. (**B**) Effect of *zag-1*, *dat-1*, *ser-7*, or *dop-1* mutation on survival of *C. elegans* after *C. albicans* infection. Statistical comparisons of the survival plots indicate that, after *C. albicans* infection, the survival of *dat-1(ok157)* or *ser-7*(tm325) was significantly (*p* < 0.0001) different from that of wild-type. Three replicates were performed. (**C**) Effect of *dat-1* or *ser-7* mutation on CFU of *C. albicans* in *C. elegans*. *C. elegans* were infected with *C. albicans* lawns for 24 h. (**G**) Effect of *dat-1* or *ser-7* mutation on expression levels of putative antimicrobial genes in *C. elegans* after *C. albicans* infection. *C. elegans* were infected with *C. albicans* lawns for 24 h. The reactions were performed in triplicate. Bars represent mean ± SD. **p < 0.01 vs wild-type.

In *C. elegans*, SER-7, DAT-1, ZAG-1, EGL-3, DOP-1, and USP-46 might act as the downstream targets of GLR-1 in regulating different biological events [34-39]. After *C. albicans* infection, *glr-1* mutation did not significantly affect the transcriptional expressions levels of *egl-3* and *usp-46* (Fig. 7A). By contrast, after *C. albicans* infection, *glr-1* mutation significantly decreased the transcriptional expression levels of *ser-7*, *zag-1*, and *dop-1*, and increased the transcriptional expression of *dat-1* (Fig. 7A). After *C. albicans* infection, we further observed that although *zag-1* or *dop-1* mutation did not significantly affect survival, *dat-1* mutation significantly increased survival and *ser-7* mutation significantly decreased survival (Fig. 7B). In *C. elegans*, *ser-7* encodes a metabotropic serotonin receptor, and *dat-1* encodes a dopamine transporter. Moreover, *dat-1* mutation significantly decreased the CFU of *C. albicans*, whereas *ser-7* mutation significantly increased the CFU of *C. albicans* in *C. elegans* (Fig. 7C). Additionally, *dat-1* mutation significantly increased the expression levels of *abf-2*, *cnc-4*, and *cnc-7*; however, *ser-7* mutation significantly decreased the expression levels of *abf-2*, *cnc-4*, *cnc-7*, and *fipr-22/23* (Fig. 7D). These results implied that DAT-1 and SER-7 might act as the potential targets of GLR-1 in the regulation of innate immune response to fungal infection.

### Genetic Interaction between GLR-1 and SER-7 in the Regulation of Innate Immune Response to *C. albicans* Infection

**Fig. 8:**
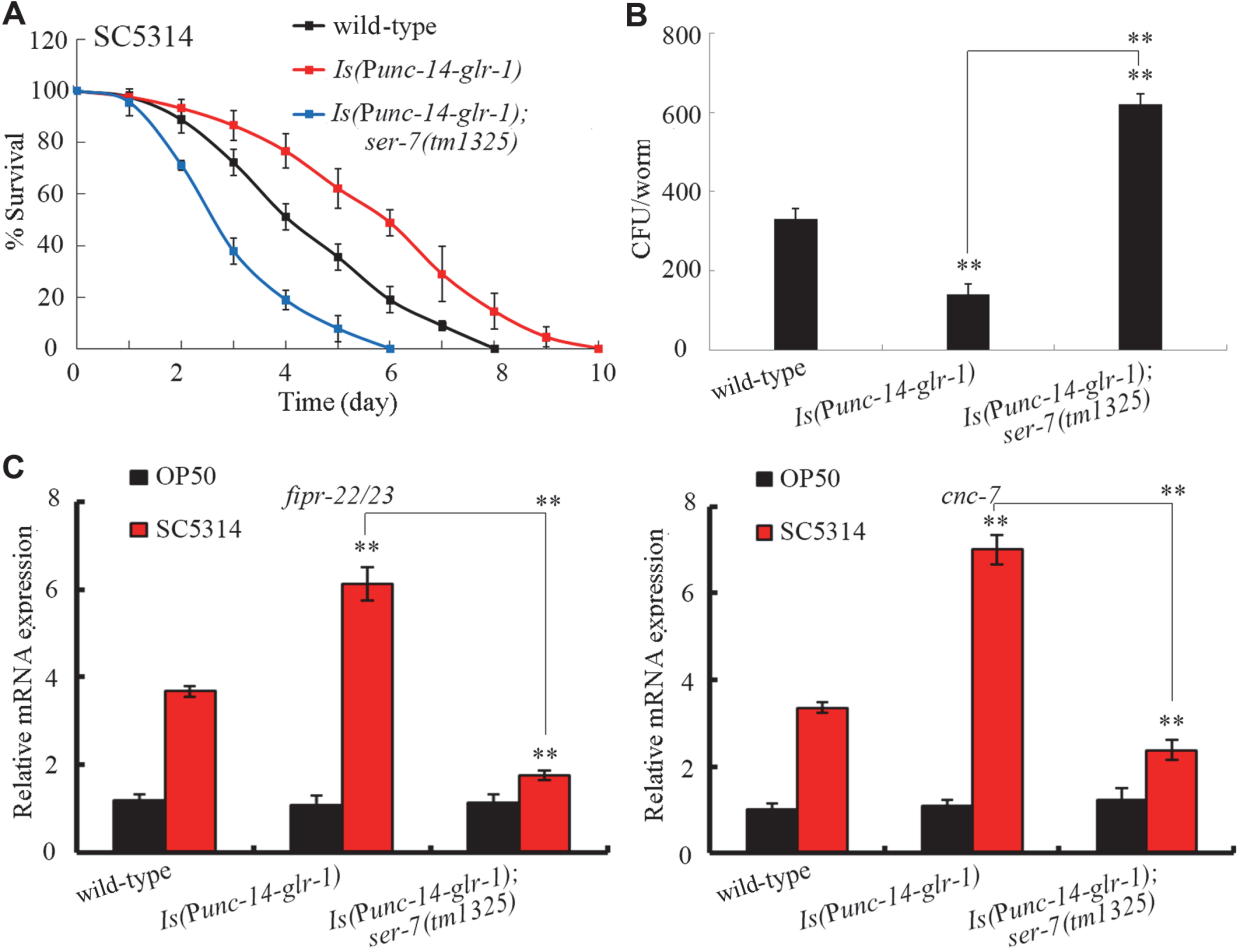
Genetic interaction between GLR-1 and SER-7 in the regulation of innate immune response to *C. albicans* infection. (**A**) Genetic interaction between GLR-1 and SER-7 in regulating survival of *C. elegans* after *C. albicans* infection. Statistical comparisons of the survival plots indicate that, after *C. albicans* infection, the survival of *Is*(P*unc*-14-*glr-1*) was significantly (*p* < 0.0001) different from that of wild-type, and the survival of *Is*(P*unc*-14-*glr-1*);*ser-7(tm1325)* was significantly (*p* < 0.0001) different from that of *Is*(P*unc*-14-*glr-1*). Three replicates were performed. (**B**) Genetic interaction between GLR-1 and SER-7 in regulating CFU of *C. albicans* in *C. elegans*. *C. elegans* were infected with *C. albicans* lawns for 24 h. (**C**) Genetic interaction between GLR-1 and SER-7 in regulating expression levels of putative antimicrobial genes in *C. elegans* after *C. albicans* infection. *C. elegans* were infected with *C. albicans* lawns for 24 h. The reactions were performed in triplicate. Bars represent mean ± SD. **p < 0.01 vs wild-type (if not specially indicated).

We generated the transgenic strain of *Is*(P*unc*-14-*glr-1*) overexpressing the neuronal GLR-1 to examine the genetic interaction between GLR-1 and SER-7 in the regulation of innate immune response to fungal infection. After *C. albicans* infection, the neuronal overexpression of GLR-1 significantly increased survival (Fig. 8A), and decreased the CFU of *C. albicans* in *C. elegans* (Fig. 8B). Additionally, after *C. albicans* infection, the neuronal overexpression of GLR-1 significantly increased the expression levels of *cnc-7* and *fipr-22/23* (Fig. 8C), whereas the overexpression of neuronal GLR-1 did not significantly affect the expression levels of *abf-2* and *cnc-4* (data not shown). Moreover, we found that *ser-7* mutation significantly decreased survival, increased the CFU of *C. albicans*, and suppressed the expression levels of *cnc-7* and *fipr-22/23* in the transgenic strain of *Is*(P*unc*-14-*glr-1*) (Fig. 8). Therefore, *ser-7* mutation might suppress the innate immune response of the transgenic strain of *Is*(P*unc*-14-*glr-1*) to fungal infection. That is, SER-7 acted as the downstream target of neuronal GLR-1 in the regulation of innate immune response to fungal infection.

### Genetic Interaction between GLR-1 and DAT-1 in the Regulation of Innate Immune Response to *C. albicans* Infection

**Fig. 9:**
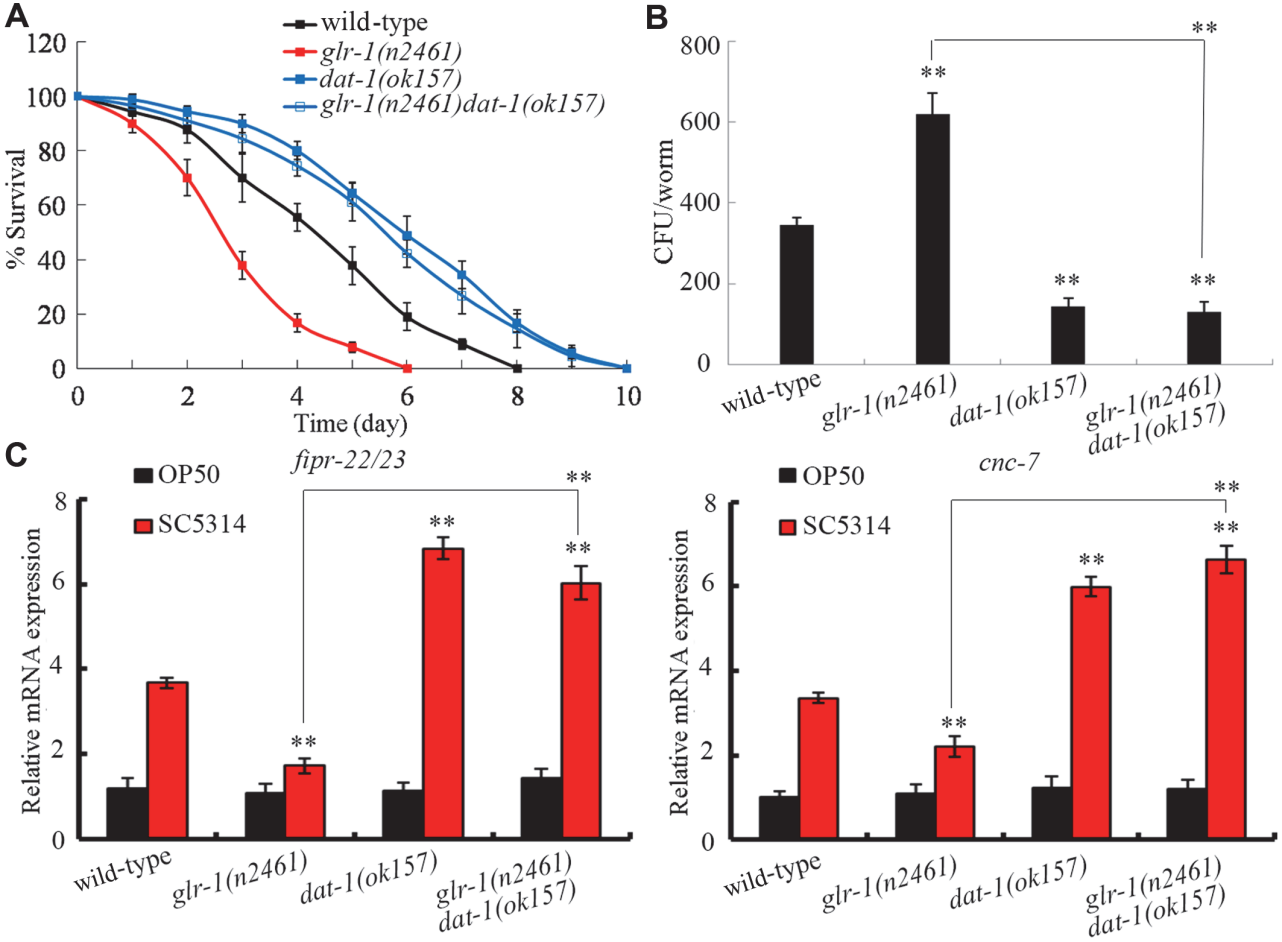
Genetic interaction between GLR-1 and DAT-1 in the regulation of innate immune response to *C. albicans* infection. (**A**) Genetic interaction between GLR-1 and DAT-1 in regulating survival of *C. elegans* after *C. albicans* infection. Statistical comparisons of the survival plots indicate that, after *C. albicans* infection, the survival of *glr-1(n2461)dat-1(ok157)* was significantly (*p* < 0.0001) different from that of *glr-1(n2461)* mutant. Three replicates were performed. (**B**) Genetic interaction between GLR-1 and DAT-1 in regulating CFU of *C. albicans* in *C. elegans*. *C. elegans* were infected with *C. albicans* lawns for 24 h. (**C**) Genetic interaction between GLR-1 and DAT-1 in regulating expression levels of putative antimicrobial genes in *C. elegans* after *C. albicans* infection. *C. elegans* were infected with *C. albicans* lawns for 24 h. The reactions were performed in triplicate. Bars represent mean ± SD. **p < 0.01 vs wild-type (if not specially indicated).

In *C. elegans*, *dat-1* is expressed in the neurons [40]. We generated the double mutant of *glr-1(n2461)dat-1(ok157)* to examine the genetic interaction between GLR-1 and DAT-1 in the regulation of innate immune response to fungal infection. We found that after *C. albicans* infection, *dat-1* mutation significantly increased survival (Fig. 9A), decreased the CFU of *C. albicans* (Fig. 9B), and increased the expression levels of *cnc-7* and *fipr-22/23* in the *glr-1(n2461)* mutant (Fig. 9C). Therefore, GLR-1 acted as the upstream target of DAT-1 and antagonized its function in the regulation of innate immune response to fungal infection.

## Discussion

Previous studies have suggested the important function of SHN-1 in the regulation of defecation rhythm behavior, pharyngeal pumping, and male fertility in *C. elegans* [25, 41]. Meanwhile, the RNAi knockdown of *shn-1* did not induce lethality or developmental abnormality [25]. In this study, we further observed that, after *C. albicans* infection for 6, 12, or 24 h, the transcriptional expression of *shn-1* and the expression of SHN-1::GFP were significantly increased (Fig. 1). In *C. elegans*, the loss-of-function mutation of *shn-1* increased susceptibility to *C. albicans* infection as indicated by the reduction in survival (Fig. 2A). Additionally, the loss-of-function mutation of *shn-1* increased the CFU of *C. albicans* in *C. elegans* (Fig. 2B). These results implied that after *C. albicans* infection, SHN-1 might be activated to mediate a protective mechanism in *C. elegans* against the adverse effects of *C. albicans* infection. Moreover, we observed that the transcriptional expression of *shn-1* and the expression of SHN-1::GFP were significantly decreased after *C. albicans* infection for 48 h (Fig. 1). These observations implied at least two possibilities. One possibility is that long-term *C. albicans* infection might cause abnormality in defecation behavior and pharyngeal pumping under induction by the reduction in SHN-1. Another possibility is that the protective function of increased SHN-1 expression might be insufficient to further counteract the adverse effects of long-term *C. albicans* infection.

With the aid of putative antimicrobial genes, we found that the loss-of-function mutation of *shn-1* might suppress the innate immune response of *C. elegans* to *C. albicans* infection (Fig. 2C). After *C. albicans* infection, the *shn-1* mutant exhibited the decreased expressions levels of some putative antimicrobial genes (*cnc-4*, *cnc-7*, and *fipr-22/23*) compared with wild-type *C. elegans* (Fig. 2C). Moreover, the neuronal or intestinal overexpression of SHN-1 significantly increased the expression levels of these putative antimicrobial genes after *C. albicans* infection (Fig. 4C). In this study, we further found that the tissue-specific activity of SHN-1 in the neurons or in the intestine was required for the regulation of innate immune response to *C. albicans* infection (Fig. 3). By contrast, we did not detect the function of SHN-1 in the pharynx in the regulation of innate immune response to fungal infection (Fig. 3). Previous studies have suggested that the mutations of genes encoding SHANKs are closely associated with the occurrence of ASDs, such as autism or Asperger syndrome [30, 42, 43]. Our results further implied that neuronal SHN-1/SHANK might be required for the regulation of neuronal development and innate immunity. Moreover, our results also suggested the important function of intestinal SHN-1/SHANK in the regulation of innate immune response to fungal infection.

In mammals, the SHANK protein acts as a scaffold molecule and potentially interacts with some other proteins [30]. In *C. elegans*, among the homologues of proteins interacting with SHANK, we found that only GLR-1, EGL-19, and MGL-2 might function as the potential downstream targets of SHN-1 in the regulation of innate immune response to *C. albicans* infection. After *C. albicans* infection, the mutation of *shn-1* altered the expression levels of *glr-1*, *egl-19*, and *mgl-2* (Fig. 5B). Moreover, the mutation of *glr-1*, *egl-19*, or *mgl-2* induced alterations in survival, the CFU of *C. albicans*, and the expression levels of putative antimicrobial genes in *C. albicans*-infected *C. elegans* (Figs. 5C, 5F, and 5G). Although the mutation of *shn-1* also altered the expression levels of *dyn-1* and *spc-1* in *C. albicans*-infected *C. elegans* (Fig. 5B), the mutation of *dyn-1* or the RNAi knockdown of *spc-1* did not obviously affect the survival of *C. elegans* after *C. albicans* infection (Figs. 5C and 5D).

In mammals, SHANK can mediate the proper localization of glutamate receptors [23]. Ganor et al. found that AMPA ionotropic glutamate receptor (GluR3) is expressed in high levels in normal human T cells, human T leukemia cells, and mouse anti-myelin basic protein T cells [24]. The expression of GluR3 on T cells is necessary to induce T cell activation and modulate immune function [24]. In this study, we identified GLR-1, an AMPA ionotropic glutamate receptor and homologs of GluR3 in mammals [38], as a downstream target of neuronal SHN-1 in the regulation of innate immune response to *C. albicans* infection (Fig. 6). Nevertheless, we found that the mutation of *shn-1* did not significantly affect the expression of *glr-2*, which encodes another AMPA ionotropic glutamate receptor (Fig. 5A). Additionally, the neuronal overexpression of SHN-1 did not significantly influence the expression of *mgl-2* after *C. albicans* infection (Fig. 6A). *mgl-2* encodes a metabotropic glutamate receptor in *C. elegans* [44]. These results suggested that neuronal SHN-1 could only act upstream of a certain glutamate receptor to regulate innate immune response to fungal infection.

We identified the plasma membrane dopamine transporter DAT-1[45] and the metabotropic serotonin receptor SER-7 [46] as the potential targets of GLR-1 in GLR-1-mediated signaling, which is involved in the control of innate immune response to fungal infection. The dysfunction of dopaminergic neurotransmission has been implicated in infection with the human immunodeficiency virus [47]. The homolog of SER-7 (serotonin type 7 receptor) in mammals has been discovered in human and rat immune tissues and has an important role in immune activation [48, 49]. Serotonin affects immune regulation including innate and adaptive immune system regulation [49]. In our study, after *C. albicans* infection, *glr-1* mutation altered the expression levels of *dat-1* and *ser-7*, and *dat-1* or *ser-7* mutation obviously influenced the fungal infection and innate immune response of *C. elegans* (Fig. 7). During the control of innate immune response to fungal infection, GLR-1 could positively regulate the function of SER-7 given that *ser-7* mutation inhibited the resistance and innate immune response of transgenic strain *Is*(P*unc*-14-*glr-1*) to fungal infection (Fig. 8). Moreover, GLR-1 could also negatively regulate the function of DAT-1, given that *dat-1* mutation suppressed susceptibility and increased the innate immune response of the *glr-1* mutant to fungal infection (Fig. 9). Therefore, GLR-1-mediated serotonin signaling might regulate innate immune response of animals to fungal infection by differentially affecting the functions of SER-7-mediated serotonin signaling and DAT-1-mediated dopamine signaling.

In this study, although MGL-2 did not act as the downstream target of neuronal SHN-1 in the regulation of innate immune response to fungal infection (Fig. 6A), MGL-2 might function as an important downstream target of intestinal SHN-1 in regulating innate immune response to fungal infection. That is, neuronal SHN-1 and intestinal SHN-1 might regulate innate immune response to fungal infection via a different glutamate receptor.

**Fig. 10:**
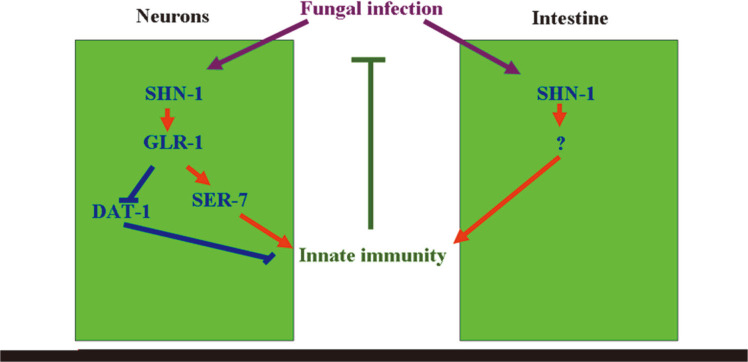
A diagram showing the molecular basis for SHN-1 in the regulation of innate immune response to fungal infection.

In conclusion, here, we examined the role of SHN-1/SHANK in the regulation of innate immune response to fungal infection and its underlying molecular mechanism. Our results suggested that the mutation of *shn-1* increased susceptibility to fungal infection and decreased innate immune response to fungal infection. Transcriptional expression of *shn-1* and the expression of SHN-1::GFP were increased after *C. albicans* infection for 6, 12, or 24 h. In *C. elegans*, SHN-1 could act in the neurons and intestine to regulate innate immune response to fungal infection. In the neurons, we identified a signaling cascade of SHN-1/SHANK-GLR-1-SER-7/DAT-1 in the regulation of innate immune response to fungal infection (Fig. 10). Our results suggested the important function of activated SHN-1 in the neurons or the intestine in protecting *C. elegans* from the adverse effects of fungal infection. Given the conservation of general defense strategies, this study also will help us understand human innate immunity against fungal infection.

## Supplemental Materials



Supplementary data for this paper are available on-line only at http://jmb.or.kr.
