# Supplementary material for: Regulation of Innate Immune Response to Fungal Infection in Caenorhabditis elegans by SHN-1/SHANK
Source: J Microbiol Biotechnol. 2020 Sep 11;30(11):1626–39. doi: 10.4014/jmb.2006.06025 (PMC9728204; doi:10.4014/jmb.2006.06025)
Supplement: Supplementary file 1 [file JMB-30-11-1626-supple.pdf]

https://doi.org/10.4014/jmb.2006.06025

Supporting Information:

Table S1. Primers for real-time PCR of antimicrobial genes

Gene	Forward primer (5'-3')	Reverse primer (5'-3')
abf-2	TGGTAATGCACAACCCCTGA	TTCGTCCGTTCCCTTTTCCT
cnc-4	GCTTCGCTACATTCTCGTCCT	GTATCCACCACCATACCCGC
cnc-7	GGACGGTACATTCCCATACC	CAGGTTCAATGCAGTATGGCTATGG
fipr-22/23	GCTGAAGCTCCACACATCC	TATCCCATTCCTCCGTATCC
tba-1	TCAACACTGCCATCGCCGCC	TCCAAGCGAGACCAGGCTTCAG
spc-1	ACGCCCTCTGGGATAAGC	TTCAAACGATGCCAAGCA
stn-1	TATTCGGGAACGAGTTAG	CACTATTCGTAAGCGTC
egl-19	AGCCATCTATCAACCCTA	TCCGCAGAATAACTCTAA
mgl-2	CTTCGTGTTGTGCCATCT	TCTATCAGCCCATCCATC
egl-8	GCAGAGGGATAGCATTCA	TCTTGTGGCACTGGACTT
ephx-1	GCAAAGGGTCACAAGATA	AGGAAGACATGAAGGGTT
abi-1	TTCTTGACGGAATAGGGC	GGAAGTGGGAATGTTGGT
dyn-1	GACTTGATGGACGAGGGA	AACCAGCTTTGCAGAGGA
atn-1	GAGTTCCGTGCGTCATTC	GGAGCCATTCGTGCCATA
gap-2	GATAATGGCGTCTTTCAC	TAGCCTCTTACGGTTTCT
glr-1	GGAAGCTGGGATGGAATG	AGCAGAAGATGCGATACG
glr-2	ATGCCTCTTATCGACTGG	TACATTTACGGGTATTGG
glr-4	AGGCTCACTTTGGACATT	ACATCCGTTCATACATCG
let-413	GGACCTCCCTGACACGAT	CACCAAGAAATGCTCCTC
pix-1	GGAATGGTGCTCCCTGAA	AGACGACGGCGTGCTTTT
F42H10.3	CACTGTTTGCGGAATGAC	AGTTGGGTTTGGTGAGAT
egl-3	AGAGGGAGGTGGACTGGAAG	ATCCTCGCTTTGTACGGGTG
zag-1	GTCTACCATCAGACGAGGCG	GGGGAGTTTGCAATTGGAGC
dat-1	CTTGCCTGGGGGCTTCATTA	AACGTGAGAGTTCGGTGCAT
ser-7	TGCTAGCACTGTGGTTAGGC	TTGCGTCGAGATAGAAGCGG
usp-46	AGACGAGGTGTTTGAGTT	TTGAGGAGGTTTCTTGAT

dop-1	CAAACATCAGCGATCAGAAA	TTGAATGCTCGTCTAAAGTC
daf-16	GAGGGAGATGAGCACAAAAA	GTGGATTCCTTCCTGGCTT
goa-1	CCATGTCACAGGAAGAGCGT	TAAGTTGCTCATGGCTCGCA
vab-3	TGCGATCAATCGCTAATACGAA	GCCGGATGATCGCTTGTTTCTC
shn-1	GCAAAGAGGCGGAGTAAA	GACGACGAGGCAACAGAC

Table S2. Primers for DNA constructs

Gene	Forward primer (5’-3’)	Reverse primer (5’-3’)
P*ges-1*	ATATCTAGAAGCCACTCAGCCACTTCA	ATAGGATCCCATCTGAATTCAAAGATA
P*unc-14*	ACGAAGCTTTTCCCAACTGGCAATACT	ATACTGCAGCCACAAAAGTTGAGAGCA
P*myo-2*	CCCAAGCTTGGTGGTGGACAGTAACTGTCTGT	CGCTCTAGACATTTCTGTGGTCTGACGATCGA
shn-1/C33B4.3c	ATCCCCGGGATGAATCAAGAGGAGGAC	ATACCATGGTCACTGGAGGAGGCCACG
daf-16/R13H8.1a	ATACCCGGGATGAACGACTCAATAGAC	CGCGGTACCTTACAAATCAAAATGAAT
pmk-1/B0218.3	ATCCCCGGGATGTTTCCACAGACAACA	CTGGGATCCCTACGATTCCATTTTCTC
glr-1/C06E1.4	ATACCCGGGATGTTTTCTTCGTTTTCT	GACGGTACCTCAGACAGCTGTGTTGTA
